# Regional disparities and dynamic evolution of suicide prevention and intervention efficiency in Japan

**DOI:** 10.3389/fpubh.2024.1359902

**Published:** 2025-01-07

**Authors:** Yin Tang

**Affiliations:** Graduate School of Economics, Keio University, Tokyo, Japan

**Keywords:** suicide prevention, suicide intervention, Efficiency, three-stage data envelopment analysis, Luenberger productivity index

## Abstract

**Introduction:**

This study investigates the cost-effectiveness of suicide prevention and intervention (SPI) efforts by prefectural governments in Japan. It represents the first application of a public sector efficiency evaluation model to assess government SPI initiatives. The research aims to identify spatial disparities and dynamic evaluation in SPI efficiency, providing actionable insights for policymakers.

**Methods:**

We employed a three-stage Modified Slacks-Based Measure of Super Efficiency to evaluate the SPI implementation efficiency of prefectural governments. This approach accounted for exogenous environmental and stochastic factors to isolate actual managerial efficiency. Additionally, the Luenberger productivity index was utilized to analyze the changes in SPI efficiency over time, focusing on the contributions of technological advancements and efficiency improvements.

**Results:**

The analysis revealed significant spatial disparities in SPI efficiency across prefectural governments. However, a substantial portion of these differences was attributable to exogenous environmental and stochastic factors, indicating relatively limited variations in actual managerial efficiency. The Luenberger productivity index indicated an overall upward trend in SPI productivity, driven primarily by technical change. Conversely, the analysis highlighted a decline in efficiency changes, predominantly due to reduced scale efficiency.

**Discussion:**

The findings underscore the importance of considering external environmental and stochastic factors when evaluating SPI efficiency. While technical advancements have positively influenced SPI productivity, policymakers should address the deteriorating trend in scale efficiency changes to ensure sustainable improvements in efficiency. Strategies that balance technical change and efficiency enhancements are essential for optimizing local SPI efforts.

## 1 Introduction

Although suicide prevention is a complex challenge, it is preventable through the formulation of effective public policies and the implementation of targeted interventions (1). In 2019, the Seventy-second World Health Assembly passed the World Health Organization’s (WHO) first Mental Health Action Plan. Suicide prevention is a critical component of this plan, with the goal of reducing suicide rates by one-third by 2030 (2). The plan’s development was inspired, in part, by Japan’s success in suicide prevention efforts. Since the early 1990s, Japan’s suicide rate has increased, reaching its peak in 1998, and has since remained around 30,000 individuals annually (3). To alleviate the ongoing rise in the suicide rate, the Japanese government has implemented a series of suicide prevention and intervention (SPI) measures, including encouraging local governments and municipalities to play an active role in SPI while providing financial support for related activities.

Many scholars attribute the sustained decrease in suicide rates, particularly among males, after 2009 to the allocation of funds from the Regional Comprehensive Suicide Prevention Emergency Strengthening Fund, or Regional Fund (RF) (4–6). Between 2009 and 2014, local prefectural governments received 18.35 billion Japanese yen from the RF to support SPI activities among local public organizations^1^ (7). This allocation effectively granted prefectural governments discretion in fund utilization. Specifically, based on SPI implementation plans and declared amounts submitted by municipal governments under their jurisdiction, prefectural governments formulated SPI implementation plans and applied for funds from the RF. As per the RF management guidelines, prefectural governments were responsible for fund allocation and oversight of fund utilization by subordinate public entities (8).

However, the severe financial burden and imperfect policy effectiveness have raised doubts regarding the cost-effectiveness of RF. They advocate for government efforts to improve regional SPI plans for greater cost-effectiveness (9) and propose the need for more detailed observations to establish an evidence-based and cost-effective fiscal budget structure for SPI (6). Therefore, assessing the policy effectiveness and cost-effectiveness of local governments in SPI would aid Japan’s central government in overseeing local government policy execution efficiency and providing references for future policy formulation.

As such, this study aims to analyze the cost-effectiveness of RF in local Japanese governments to understand the dynamic evolution of local SPI efficiency. The primary contributions are as follows: Firstly, surpassing existing research that overly emphasizes government outcomes in SPI while overlooking cost-effectiveness, this study utilizes input–output efficiency measurement models to assess the efficiency of SPI by local governments. It takes into account environmental and luck factors’ influence on the efficiency of local government policy implementation, filtering out relatively objective SPI efficiency using the three-stage Super Efficiency (SE) and Modified Slacks-Based Measure (MSBM) model framework (In the following text, it is abbreviated as SE-MSBM). The analysis demonstrates significant differences in SPI efficiency among local governments, where these disparities are partially attributed to environmental and luck factors, and the efficiency differences resulting from differences in local government management are exaggerated. Secondly, this paper dynamically decomposes the factors contributing to the evolution of SPI efficiency from the perspectives of productivity changes, which can be decomposed into efficiency changes and technological changes. Over time, the study confirms an overall improvement in SPI productivity, but with a declining marginal improvement, resulting from joint progress in efficiency and technological changes. Moreover, there is a tendency for productivity improvement to excessively rely on technological progress while relatively neglecting efficiency improvement, particularly in pure efficiency. As a policy recommendation, based on the research findings, we advocate for recognizing the positive role of external environmental improvements in the cost-effectiveness of SPI. Furthermore, local governments should reduce reliance on technological progress and strive to promote SPI productivity through efficiency improvement, especially in pure efficiency.

The rest of this paper is structured as follows: Section two reviews the literature related to SPI and the efficiency measurement of public sector. Section three outlines the model-building process. Section four introduces the data sources and the analytical results of the model. Section five discusses the model analysis results from section four and provides relevant policy recommendations. The final section draws conclusions based on the entire discourse presented in the paper.

## 2 Literature review

The decline in Japan’s suicide rates post-2009 owes significantly to proactive interventions at national, local government, and community levels. At the national level, policies such as the 2006 Basic Act for Suicide Prevention, the 2007 General Principles of Suicide Prevention Policy, and a series of fiscal support policies (including RF) have been proven effective in some studies (4, 5, 10). However, some studies suggest its actual policy impact has been notably limited (11). Regarding central fiscal support and SPI, research falls into two categories. One focuses on government investment in public health and welfare, along with redistribution policies and their correlation with suicide behavior (12, 13). For instance, Shiroyama, Fukuyama (13) demonstrated a correlation between reduced suicide death rates and expenditure in “public health,” “police,” “ambulance/firefighting,” “welfare,” and “education” sectors in Japan from 2009 to 2018. The second category emphasizes the significance of central fiscal support for local SPI efforts (5, 6, 10, 14). Nakanishi, Yamauchi (10) highlighted that national fund facilitated the establishment of community SPI systems, streamlining the implementation of local SPI projects.

The WHO noted that besides evidence on the effect or impact of suicide prevention strategies, health planners and policymakers also need to understand the expected costs and cost-effectiveness to ensure that these strategies are economically viable. However, there is still a global shortage of robust economic research on the cost-effectiveness of suicide prevention (1). In the case of Japan, the focus on the cost-effectiveness of local government suicide intervention policies (SPI) stems partly from strained fiscal conditions (5, 6) and partly from the unstable and variable effectiveness of SPI measures. Yonemoto, Kawashima (14) found that although almost all local governments offered gatekeeper training programs, there was a lack of assessment of policy outcomes. Kato and Okada (5), analyzing prefectural data, noted variations in the policy impact of RF on male and female suicide rates. Yet, scarce objective studies have quantitatively evaluated the cost-effectiveness of national or local government executions of SPI policies in Japan.

The WHO categorizes the risk factors influencing suicide rates into several levels: health system, society, community, relationships, and individual, and provides corresponding intervention measures (although these are not exhaustive) (1). Similarly, Stack (15) categorizes influences on suicide into political, social, cultural, and economic dimensions. From a political perspective, increased social welfare and public assistance often correlate with reduced suicide rates (15, 16). Socially, marital and parental social institutions remain protective factors against suicide, albeit with gender disparities in their protective effects (17, 18). Additionally, most articles support the view that social isolation causally relates to suicide, while social support serves a protective function against suicide (19). Some studies indicate a correlation between alcohol consumption issues and suicide rates (15, 20, 21). Culturally, religious beliefs often negatively correlate with suicide rates, but mediating factors require further exploration (15). Moreover, Japan’s traditional notion of honorable suicide and modern conservative attitudes toward suicide are considered reasons for high suicide rates (22). Lastly, economically, a sluggish economic environment correlates with high suicide rates. Specifically, high unemployment rates and low job availability elevate suicide rates, especially among middle-aged men (22, 23). However, some research contests the direct causality between unemployment rates and suicide rates, at least concerning Japan (24). Additionally, macroeconomic indicators like regional GDP and CPI are believed to influence suicide rates (25, 26). Therefore, to derive relatively objective management efficiency in evaluating local government efficiency, environmental factors influencing the implementation of local government policies need consideration.

Assessing public sector policy efficiency has been a widely debated topic in academia. However, scarcely any studies have delved into efficiency assessments of SPI policies. With respect to the possible reasons, on one hand, efficiency analyses in healthcare sectors primarily focus on healthcare facility (hospitals, primary healthcare facilities) efficiency, with few studies exploring system-level (national or sub-national) efficiency (27). On the other hand, some studies suggest inconsistencies in the effectiveness of SPI policies across countries, with limited evidence supporting their impact on reducing suicide rates (11, 28, 29). Nevertheless, in Japan, there’s a prevalent belief in academia that the implementation of RF is causally linked to the decline in suicide rates, particularly among males (5, 6, 10).

## 3 Materials and methods

This study can be divided into two parts. The first part involves estimating the SPI efficiency of each prefecture using a three-stage SE-MSBM model. The second part calculates the Luenberger total factor productivity (LTFP) index based on the estimated SPI efficiency to reflect the dynamic evolution of this efficiency.

### 3.1 Three-stage SE-MSBM model

The Slacks-Based Measure is a type of Data Envelopment Analysis (DEA), which is a is a non-parametric method used to evaluate the efficiency of decision-making units (DMUs) by constructing a piecewise linear frontier against which all observations are compared. It uses slack variables to evaluate the efficiency of DMUs. This method was proposed by Tone (30), who also introduced a way to assess the SE of these units. However, traditional Slacks-Based Measure struggles with translation invariance. This can cause distortions when assessing DMUs with negative inputs or outputs. To resolve this, Sharp, Meng (31) developed the MSBM. This method is tailored for systems that naturally include negative values.

**Figure 1:**
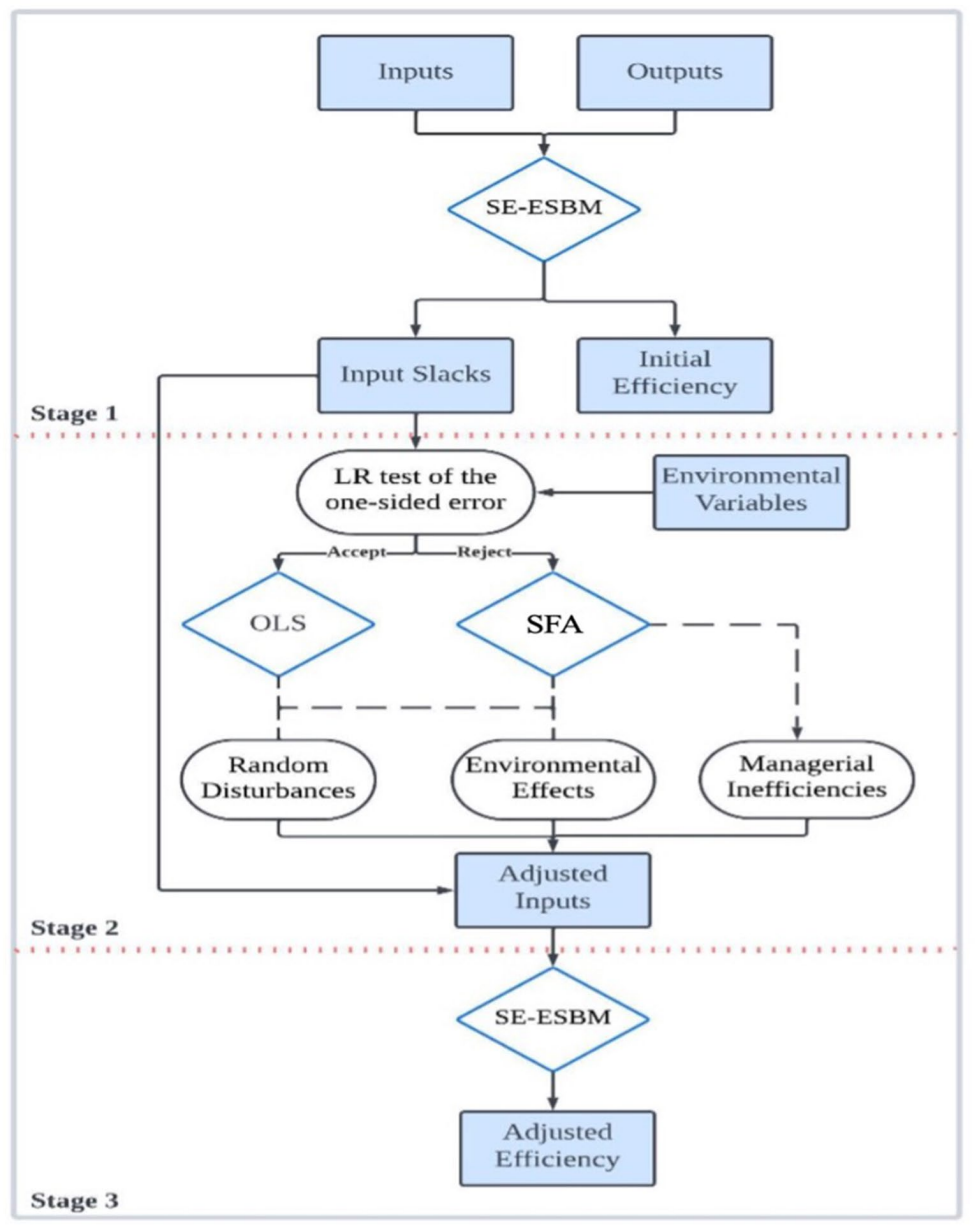
Methodological framework of the three-stage SE-MSBM model.

The three-stage SE-MSBM model builds on the existing three-stage DEA framework. It replaces the traditional DEA model with the SE-MSBM. This model, proposed by Fried and Lovell, improves on earlier DEA versions. The three-stage DEA method proposed by Fried and Lovell (32). It overcomes the limitations of the one-stage DEA method, which cannot measure factors affecting efficiency, and the two-stage DEA method, which assumes a given form of influencing factor functions and cannot eliminate environmental impact factors. The main goal of this model is to filter out environmental effects and statistical noise from managerial inefficiency. This makes the efficiency assessments of DMUs more precise. For a detailed methodological explanation, refer to Figure 1. It provides a comprehensive framework for the three-stage SE-MSBM model.

#### 3.1.1 Stage 1: initial MSBM evaluation of DMUs performance

The standard form of efficiency score evaluated by MSBM is given as follows:


(1) $$\begin{array}{l} \min\rho =\frac{1-\frac{1}{m}\sum_{i=1}^{m}\frac{s_{i}^{-}}{R_{i0}^{-}}}{1+\frac{1}{q}\sum_{r=1}^{q}\frac{s_{r}^{+}}{R_{r0}^{+}}}\\ s.t.\overset{n}{\underset{j=1,\neq 0}{\sum }}x_{ij}\lambda_{j}+s_{i}^{-}=x_{i0}\\ \overset{n}{\underset{j=1,\neq 0}{\sum }}y_{rj}\lambda_{j}-s_{r}^{+}=y_{r0} \end{array}\begin{array}{l} \overset{n}{\underset{j=1,\neq 0}{\sum }}\lambda_{j}=1\\ R_{i0}^{-}=x_{i0}-\min\left(x_{i}\right)\\ R_{r0}^{+}=\max\left(y_{r}\right)-y_{r0}\\ \lambda_{j},s_{i}^{-},s_{r}^{+}\geq 0 \end{array}$$

Here,

- jindicates the $j^{th}$ DMU in n DMUs.
- $x_{i}$and$y_{r}$ represent the $i^{th}$ input variable in m input variables and the $r^{th}$ output variable in q variables, respectively.
- $R_{i0}$ and $R_{r0}$ represent the range of possible improvement for the corresponding inputs and outputs. λ denotes the weight vector. Additionally, if $R_{i0}^{-}=0$, the respective $\frac{s_{i}^{-}}{R_{i0}^{-}}$ term is removed from the objective function, and if $R_{r0}^{+}=0$, the corresponding $\frac{s_{i}^{+}}{R_{r0}^{+}}$ term is removed from the objective function.

For the $DMU\left(x_{0},y_{0}\right)$ that is efficient under the SBM, we define its super-efficiency as the optimal objective function value $\rho^{*}$ given by:


(2) $$\begin{array}{l} \rho^{*}=\min\rho =\frac{\frac{1}{m}\sum_{i=1}^{m}\frac{{\bar{x}}_{i}}{R_{i0}^{-}}}{\frac{1}{q}\sum_{r=1}^{q}\frac{{\bar{y}}_{r}}{R_{r0}^{+}}}\\ s.t.\bar{x}\geq \overset{n}{\underset{j=1,\neq 0}{\sum }}\lambda_{j}x_{j}\\ \bar{y}\geq \overset{n}{\underset{j=1,\neq 0}{\sum }}\lambda_{j}y_{j} \end{array}\begin{array}{l} \overset{n}{\underset{j=1,\neq 0}{\sum }}\lambda_{j}=1\\ R_{i0}^{-}=x_{i0}-\min\left(x_{i}\right)\\ R_{r0}^{+}=\max\left(y_{r}\right)-y_{r0}\\ \bar{x}\geq x_{0},\bar{y}\leq y_{0}\;\mathrm{and}\;\lambda \geq 0. \end{array}$$

In this stage, we conducted a simple SE-MSBM under the assumption of variable returns to scale (VRS) and obtained the efficiency values of each DMU without excluding environmental factors and the slack variables of each input variable. The former will be used for subsequent comparative analysis, while the latter will be included as dependent variables in the second stage.

#### 3.1.2 Stage 2: filtering out the influence of environmental effects

Based on the MSBM model from the first stage and input–output variable data, we can determine the slack values of each input and output, which represent the difference between the actual values and the optimal values. The slack value is influenced by environmental effects, statistical noise, and managerial inefficiency (32).

What we aim to do in the stage 2 is filter out the environmental effects and statistical noise, which are unrelated to managerial efficiency, from slacks. Subsequently, based on this, we adjust the original input and output indicators so that all DMUs face the same external environment and luck factors. According to Fried, Lovell (32), it is acceptable to adjust either inputs or outputs, or to simultaneously adjust both inputs and outputs. In this study, we follow the approach adopted by most scholars, adjusting only the input indicators (33–35).

To do this, we construct a Stochastic Frontier Analysis (SFA) model, as presented in Equation 3:


(3) $$s_{ijt}^{-}=f\left(Z_{ijt};\beta_{\mathrm{it}}\right)+v_{ijt}+u_{ijt}\text{,}i=1,2\ldots ,m;r=1,2\ldots ,q;j=1,2\ldots ,n;t=2010,2011\ldots 2014$$

where:

- $s_{ijt}^{-}$ is the slack value of the $i^{th}$input of the$j^{th}$ DMU at time t.
- $Z_{\mathrm{ijt}}=\left[Z_{1\mathrm{jt}}{\mathrm{,Z}}_{2\mathrm{jt}}\ldots {\mathrm{,Z}}_{\mathrm{mjt}}\right]$ represents the set of environmental variables.
- $\beta_{\mathrm{it}}$ denotes the coefficients of environmental variables to be estimated in the secondary regression equation.
- $f^{i}\left(Z_{\mathrm{ijt}};\beta_{\mathrm{it}}\right)$ are deterministic feasible slack frontiers, which capture the impact of the environmental variables on the input slack value $s_{\mathrm{ijt}}^{-}$.
- $v_{\mathrm{ijt}}+u_{\mathrm{ijt}}$ represents the error structure, where $v_{\mathrm{kit}}$ denotes random disturbances and $v\sim N\left(0,\sigma_{v}^{2}\right)$. It is assumed that the managerial inefficiency term $u_{\mathrm{ijt}}$ follows a half-normal distribution $u\sim N\left(\mu ,\sigma_{u}^{2}\;\right)$.

Before estimation, we need to specify the form of the error structure. According to Fried, Lovell (32), if the LR test of the one-sided error results fail to reject the hypothesis $\sigma_{u}^{2}=0$, it indicates that the managerial inefficiency in that input does not affect the variation in the deterministic feasible slack frontiers, and the error term comprises only random disturbances. In such a scenario, this study will utilize Ordinary Least Squares (OLS) to estimate the influence of environmental factors and use their estimated coefficients to adjust the input indicators.

Moreover, assuming a parameter $\gamma =\frac{\sigma_{u}^{2}}{\sigma_{u}^{2}+\sigma_{v}^{2}}$, which indicates the contribution of the managerial inefficiency in the variation of error. Additionally, considering whether the unit possesses time variability determines the use of either a time-invariant model or a time-varying decay model. In the time-invariant model, the $v_{\mathrm{ijt}}$ and $u_{\mathrm{ijt}}$ are independent of each other. The time-varying decay model features $u_{\mathrm{it}}=u_{i}e^{-\eta \left(t-T_{i}\right)}$, where $T_{i}$ is the last period for input i and η is the decay coefficient. Utilizing panel SFA estimation involves initially employing the time-varying decay model to estimate the equation and then conducting a test on the estimated decay parameter η. If rejecting the null hypothesis η=0, a time-varying decay model should be used; otherwise, a time-invariant model is employed for estimation.

The estimation method for the random disturbance term is based on Jondrow, Lovell (36)’s approach, as presented in Equation 4:


(4) $$E\left(u_{\mathrm{ijt}}|v_{\mathrm{ijt}}+u_{\mathrm{ijt}}\right)=\frac{\sigma \lambda }{1+\lambda^{2}}\left[\frac{\varphi \left(\frac{\varepsilon_{\mathrm{it}}\lambda }{\sigma }\right)}{\phi \left(\frac{\varepsilon_{\mathrm{it}}\lambda }{\sigma }\right)}+\frac{\varepsilon_{\mathrm{it}}\lambda }{\sigma }\right].$$

Where $\lambda =\frac{\sigma_{u}}{\sigma_{v}},\varepsilon_{\mathrm{it}}=v_{\mathrm{ijt}}+u_{\mathrm{ijt}}$. φ and ϕ respectively represent the probability density function and cumulative distribution function of the standard normal distribution.

Next, we use the estimation results of the SFA model to adjust the external environment of all DMUs to the same extent, by adjusting the input indicators through the Equation 5, aiming to eliminate the influence of heterogeneity. This adjustment ensures that the subsequent efficiency calculations are based on a homogeneous environment.


(5) $$X_{\mathrm{ijt}}^{*}=X_{\mathrm{ijt}}+\left\{\max\left[f\left(Z_{\mathrm{ijt}};{\hat{\beta }}_{\mathrm{it}}\right)\right]-f\left(Z_{\mathrm{ijt}};{\hat{\beta }}_{\mathrm{it}}\right)\right\}+\left[\max\left(v_{\mathrm{ijt}}\right)-v_{\mathrm{ijt}}\right],i=1,2\ldots ,m;j=1,2\ldots ,n;t=2010,2011\ldots 2014.$$

Where $X_{\mathrm{ijt}}^{*}$ represents the adjusted $i^{\mathrm{th}}$ input indicator of the $j^{\mathrm{th}}$ DMU at time t. After adjusting for environmental factors and random error terms, all DMUs will face the same external environment and luck factors.

#### 3.1.3 Stage 3: running MSBM again after adjusting input variables

Using the adjusted input variables $X_{\mathrm{ijt}}^{*}$ obtained from the second stage to replace the original input data $X_{\mathrm{ijt}},$ calculate the new efficiency values based on the SE-MSBM model performed in Stage 1. As the influence of environmental and random error factors has been eliminated, the efficiency scores obtained in the third stage more accurately reflect the managerial efficiency of each DMU.

### 3.2 SBM based adjacent Luenberger productivity index

At present, the Malmquist-Luenberger method stands as the most commonly used approach for measuring production indices, yet it harbors certain unavoidable limitations. On one hand, within the Malmquist-Luenberger productivity index measurement method, there’s a need to select a measurement angle either under conditions of cost minimization or profit maximization (37). On the other hand, the multiplicative form of index construction renders it incapable of calculating the Malmquist-Luenberger index when negative efficiencies exist. Luenberger proposed an alternative, addressing these issues with the LTFP index. On one hand, it allows for profit maximization usage and reduces measurement inputs without selecting a measurement perspective, thereby enhancing output (37, 38). On the other hand, even in the presence of negative efficiencies, the additive structure of LTFP index make it possible to reflect real efficiency improvements. Hence, this study opts for the LTFP index to gauge improvements in efficiency over a time series.

During period t, assuming $D_{0}^{t}\left(x_{0}^{t}{\mathrm{,y}}_{0}^{t}\right)$ represents the optimal solution for Problem 1; if $D_{0}^{t}\left(x_{0}^{t}{\mathrm{,y}}_{0}^{t}\right)$ is deemed efficient, then $D_{0}^{t}\left(x_{0}^{t}{\mathrm{,y}}_{0}^{t}\right)$ is the optimal solution for Problem 2. If $\mathrm{DMU}\left(x_{0}^{t+1}{\mathrm{,y}}_{0}^{t+1}\right)\;$is observable, similarly, assuming $D_{0}^{t+1}\left(x_{0}^{t+1}{\mathrm{,y}}_{0}^{t+1}\right)$ is the optimal solution for period t+1. Moreover, if we simply transfer inputs and outputs from period t to period t+1, the optimal solution under the reference set of DMUs in period t+1 can be denoted by the reciprocal efficiency $D_{0}^{t+1}\left(x_{0}^{t}{\mathrm{,y}}_{0}^{t}\right).$ Similarly, $D_{0}^{t}\left(x_{0}^{t+1}{\mathrm{,y}}_{0}^{t+1}\right)$represents the optimal solution of transferring inputs and outputs from period t+1 to period t within the reference set of DMUs in period t. The optimal problem of $D_{0}^{t}\left(x_{0}^{t+1}{\mathrm{,y}}_{0}^{t+1}\right)$ is given as Equation 6:


(6) $$\begin{array}{l} \min D_{0}^{t}\left(x_{0}^{t+1}{\mathrm{,y}}_{0}^{t+1}\right)=\frac{1-\frac{1}{m}\sum_{i=1}^{m}\frac{s_{i}^{-}}{R_{i0t}^{-}}}{1+\frac{1}{q}\sum_{r=1}^{q}\frac{s_{r}^{+}}{R_{r0t}^{+}}}\\ s.t.\overset{n}{\underset{j=1,\neq 0}{\sum }}x_{\mathrm{ijt}}\lambda_{j}+s_{i}^{-}=x_{i0,t+1}\\ \overset{n}{\underset{j=1,\neq 0}{\sum }}y_{\mathrm{rjt}}\lambda_{j}-s_{r}^{+}=y_{r0,t+1} \end{array}\begin{array}{l} \overset{n}{\underset{j=1,\neq 0}{\sum }}\lambda_{j}=1\\ R_{i0t}^{-}=x_{i0,t+1}-\min\left(x_{\mathrm{it}}\right)\\ R_{r0t}^{+}=\max\left(y_{\mathrm{rt}}\right)-y_{r0,t+1}\\ \lambda_{j},s_{i}^{-},s_{r}^{+}\geq 0 \end{array}$$

For the efficient $\mathrm{DMU}\left(x_{0}{\mathrm{,y}}_{0}\right)$ under the SBM, its super-efficiency as the optimal objective function value given by Equation 7:


(7) $$\begin{array}{l} D_{0}^{t}{\left(x_{0}^{t+1}{\mathrm{,y}}_{0}^{t+1}\right)}^{*}=\min D_{0}^{t}\left(x_{0}^{t+1}{\mathrm{,y}}_{0}^{t+1}\right)=\frac{\frac{1}{m}\sum_{i=1}^{m}\frac{{\bar{x}}_{i}}{R_{i0t}^{-}}}{\frac{1}{q}\sum_{r=1}^{q}\frac{{\bar{y}}_{r}}{R_{r0t}^{+}}}\\ s.t.\bar{x}\geq \overset{n}{\underset{j=1,\neq 0}{\sum }}\lambda_{j}x_{j}\\ \bar{y}\geq \overset{n}{\underset{j=1,\neq 0}{\sum }}\lambda_{j}y_{j} \end{array}\begin{array}{l} \overset{n}{\underset{j=1,\neq 0}{\sum }}\lambda_{j}=1\\ R_{i0}^{-}=x_{i0}-\min\left(x_{i}\right)\\ R_{r0}^{+}=\max\left(y_{r}\right)-y_{r0}\\ \bar{x}\geq x_{0},\bar{y}\leq y_{0}\;\mathrm{and}\;\lambda \geq 0. \end{array}$$

The aforementioned $D_{0}^{t}\left(x_{0}^{t}{\mathrm{,y}}_{0}^{t}\right)$, $D_{0}^{t+1}\left(x_{0}^{t+1}{\mathrm{,y}}_{0}^{t+1}\right)$, $D_{0}^{t}\left(x_{0}^{t+1}{\mathrm{,y}}_{0}^{t+1}\right)$, and $D_{0}^{t+1}\left(x_{0}^{t}{\mathrm{,y}}_{0}^{t}\right)$ represent the optimal solutions under the VRS assumption, i.e., subject to the constraint $\overset{n}{\underset{j=1,\neq 0}{\sum }}\lambda_{j}=1$. By removing this constraint, we obtain the optimal solutions under the assumption of constant returns to scale (CRS). For clarity, subscripts v and c are used to, respectively, indicate the optimal solutions under VRS and CRS assumptions. Additionally, it’s important to note that under the CRS assumption, in the MSBM model, the maximum potential improvement $R_{i0}$ of the evaluated ${\mathrm{DMU}}_{0}$ might exceed $s_{i}$, implying $s_{i}>R_{i0}$, which could lead to a negative value for the objective function (31). This is one of the reasons why we opt for the LTFP index to specify productivity changes.

Therefore, the LTFP index, which reflects the dynamic change of productivity in two adjacent periods can be expressed as Equation 8:


(8) $$\begin{array}{l} {\mathrm{LTFP}}_{t-1}^{t}=\frac{1}{2}\left[D_{c}^{t}\left(x^{t}{\mathrm{,y}}^{t}\right)-D_{c}^{t}\left(x^{t-1}{\mathrm{,y}}^{t-1}\right)\right]\\ +\frac{1}{2}\left[D_{c}^{t-1}\left(x^{t}{\mathrm{,y}}^{t}\right)-D_{c}^{t-1}\left(x^{t-1}{\mathrm{,y}}^{t-1}\right)\right]. \end{array}$$

The LTFP index consists of two main components: efficiency change (LEC) (Equation 9) and technical change (LTC) (Equation 10). Additionally, LEC and LTC can be decomposed into pure efficiency changes (LPEC) (Equation 11) and scale efficiency changes (LSEC) (Equation 12), as well as pure productivity technology changes (LPTP) (Equation 13) and technology scale changes (LTPSC) (Equation 14). Their formulas are as follows (37, 39):


(9) $${\mathrm{LEC}}_{t-1}^{t}=D_{c}^{t}\left(x^{t}{\mathrm{,y}}^{t}\right)-D_{c}^{t-1}\left(x^{t-1}{\mathrm{,y}}^{t-1}\right)$$

(10) $$\begin{array}{l} {\mathrm{LTC}}_{t-1}^{t}=\frac{1}{2}\left[D_{c}^{t-1}\left(x^{t}{\mathrm{,y}}^{t}\right)-D_{c}^{t}\left(x^{t}{\mathrm{,y}}^{t}\right)\right]\\ +\frac{1}{2}\left[D_{c}^{t-1}\left(x^{t-1}{\mathrm{,y}}^{t-1}\right)-D_{c}^{t}\left(x^{t-1}{\mathrm{,y}}^{t-1}\right)\right] \end{array}$$

(11) $${\mathrm{LPEC}}_{t-1}^{t}=D_{v}^{t}\left(x^{t}{\mathrm{,y}}^{t}\right)-D_{v}^{t-1}\left(x^{t-1}{\mathrm{,y}}^{t-1}\right)$$

(12) $$\begin{array}{l} {\mathrm{LSEC}}_{t-1}^{t}=\left[D_{c}^{t}\left(x^{t}{\mathrm{,y}}^{t}\right)-D_{v}^{t}\left(x^{t}{\mathrm{,y}}^{t}\right)\right]\\ -\left[D_{c}^{t-1}\left(x^{t-1}{\mathrm{,y}}^{t-1}\right)-D_{v}^{t-1}\left(x^{t-1}{\mathrm{,y}}^{t-1}\right)\right] \end{array}$$

(13) $$\begin{array}{l} {\mathrm{LPTP}}_{t-1}^{t}=\frac{1}{2}\left[D_{v}^{t-1}\left(x^{t}{\mathrm{,y}}^{t}\right)-D_{v}^{t}\left(x^{t}{\mathrm{,y}}^{t}\right)\right]\\ +\frac{1}{2}\left[D_{v}^{t-1}\left(x^{t-1}{\mathrm{,y}}^{t-1}\right)-D_{v}^{t}\left(x^{t-1}{\mathrm{,y}}^{t-1}\right)\right] \end{array}$$

(14) $$\begin{array}{l} {\mathrm{LTPSC}}_{t-1}^{t}=\frac{1}{2}\{[D_{c}^{t-1}{\mathrm{(x}}^{t}{\mathrm{,y}}^{t})-D_{v}^{t-1}(x^{t}{\mathrm{,y}}^{t})]-[D_{c}^{t}(x^{t}{\mathrm{,y}}^{t})\\ -D_{v}^{t}(x^{t}{\mathrm{,y}}^{t})]\}\\ +\frac{1}{2}\{[D_{c}^{t-1}(x^{t-1}{\mathrm{,y}}^{t-1})-D_{v}^{t-1}(x^{t-1}{\mathrm{,y}}^{t-1})]\\ -[D_{c}^{t}(x^{t-1}{\mathrm{,y}}^{t-1})-D_{v}^{t}(x^{t-1}{\mathrm{,y}}^{t-1})]\}. \end{array}$$

These indices satisfy the following relationship presented in Equation 15:


(15) $$\begin{array}{l} {\mathrm{LTFP}}_{t-1}^{t}={\mathrm{LEC}}_{t-1}^{t}+{\mathrm{LTC}}_{t-1}^{t}={\mathrm{LPEC}}_{t-1}^{t}\\ +{\mathrm{LSEC}}_{t-1}^{t}+{\mathrm{LPTP}}_{t-1}^{t}+{\mathrm{LTPSC}}_{t-1}^{t}. \end{array}$$

According to the definitions of the LTFP index and its decomposition, LEC reflects the change in relative efficiency of the DMU across different periods, known as the “catch-up effect.” On the other hand, LTC represents the movement of the efficiency frontier between periods t and t-1, denoting the “frontier shift effect.” Additionally, both LEC and LTC can be decomposed into two components: changes in pure efficiency and changes in scale efficiency.

The computation of the LTFP index requires estimation under CRS and VRS assumptions, where four are derived based on CRS and the remaining four are computed assuming VRS. In this context, LPEC, LPTP, LSEC, and LTPSC being greater than 0 (or less than 0) signify specific changes: pure technical productivity increases (or decreases), technological advancement (or regression), scale efficiency improvement (or decline), and deviation of technology from the optimal scale state of the DMU from period t to t+1.

## 4 Results

### 4.1 Variable and data selection

The study utilized data from various prefectures in Japan for a period spanning 2010 to 2014, encompassing a total of 5 years. Despite RF being established in 2009, the initial financial declaration data from each prefecture was incomplete at its inception. Therefore, we excluded the data for the year 2009. Additionally, due to the non-directional nature of the MSBM model, which does not support the efficiency calculation for DMUs with all inputs or outputs equal to zero, we omitted DMUs that had no reported RF declarations, resulting in the removal of a collective total of five observations.

#### 4.1.1 Stage 1 and stage 3: input and output indicators

The per capita amounts allocated to the five programs funded by the RF^2^: Development Program of Leaders and Listeners (DPLL), Personal Consultation Support Program (PCSP), Telephone Consultation Support Program (TCSP), Enlightenment Program (EP), Intervention Model Program (IMP) are included as input indicators in the SE-MSBM model. According to Cantor and Poh (40), the evaluation framework for the efficiency of primary healthcare institutions in the DEA model requires the inclusion of primary inputs related to labor, capital-related inputs, and inputs related to consumable resources. As per the lines for the Management and Operation of the Regional Suicide Prevention Emergency Strengthening Fund, necessary expenses, wages, compensation, social insurance premiums, travel expenses, necessary expenses, service charges, usage fees, rental fees, equipment purchase costs, commission fees, subsidies, etc., can all be withdrawn from the RF. Therefore, utilizing RF as input indicators is justified (8). Additionally, the data for RF is sourced from publicly available data provided by the Japanese Ministry of Health, Labour, and Welfare.

Regarding the output indicators, I have selected the decrease in suicide rates for both men (DSRM) and women (DSRW) compared to 2008 as two separate output variables. The suicide rate is defined as the proportion of deaths by suicide to the total population of the DMUs for the current period, which is sourced from publicly available data provided by the Japan National Police Agency (41). This data is compiled based on the location where the individuals who died by suicide were discovered, rather than their registered residential address. Therefore, the decline in the suicide rate for males and females, as output variables, can be defined as Equations 16, 17, respectively:


(16) $$\begin{array}{l} {\mathrm{DSRM}}_{\mathrm{jt}}=\frac{\mathrm{Number of}\;{\mathrm{male suicides}}_{j2008}}{{\mathrm{male population}}_{j2008}}\\ -\frac{\mathrm{Number of}\;{\mathrm{male suicides}}_{\mathrm{jt}}}{{\mathrm{male population}}_{\mathrm{jt}}} \end{array}$$

(17) $$\begin{array}{l} {\mathrm{DSRW}}_{\mathrm{jt}}=\frac{\mathrm{Number of}\;{\mathrm{female suicides}}_{j2008}}{{\mathrm{female population}}_{j2008}}\\ -\frac{\mathrm{Number of}\;{\mathrm{female suicides}}_{\mathrm{jt}}}{{\mathrm{female population}}_{\mathrm{jt}}}. \end{array}$$

**Figure 2:**
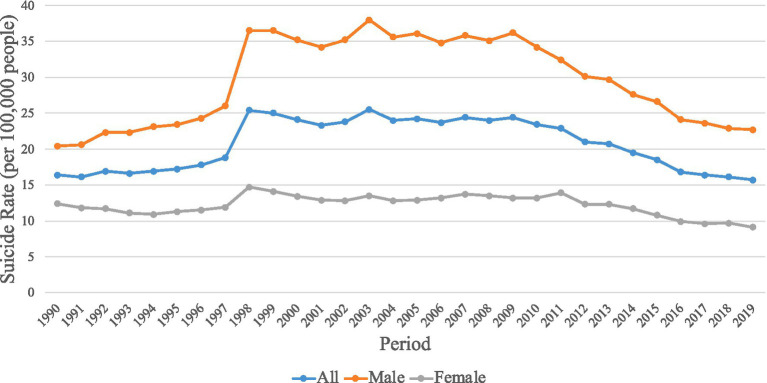
Trends in suicide rates in Japan (by gender).

The reasons for designing output indicators in this manner is as follows:

Firstly, why differentiate between genders? According to the nationwide suicide rate statistics in Japan depicted in Figure 2, over the 6 years (2009–2014) within which the RF fund was available nationwide, there was a significant overall decrease in the national suicide rate. However, the contribution to the decline in the male suicide rate was notably greater than that in the female suicide rate (7). If a DMU’s inputs from RF led to a decrease in male suicide rates but an increase in female suicide rates, using the total population’s suicide rate as an output variable could potentially result in a calculated output of zero, which would evidently be unfair. Therefore, separating the output variables by gender would better aid in evaluating the actual efficiency of the DMU.

Secondly, why use the difference from the 2008 suicide rates as inputs rather than including the suicide rate of each year as undesirable outputs in the model? As per the Guidelines for the Management and Operation of the Regional Suicide Prevention Emergency Strengthening Fund, any SPI program funded or subsidized by the national treasury implemented by each DMU before the establishment of RF, as well as personnel expenses of permanent staff in relevant administrative agencies, are not covered within the scope of RF funding. Consequently, it is assumed that before 2009, some DMUs had already been implementing SPI projects, which continued post-RF establishment. Therefore, the variations in suicide rates from 2009 onwards can be regarded as the policy effects resulting from RF input.

The detailed information regarding input and output indicators is provided in Table 1.

**Table 1:** Descriptive statistics of input and output indicators.

Indicators	*N*	Mean	SD	Max	Min
Input					
PCSP	230	4197.617	4169.657	26502.622	0
TCSP	230	3156.205	2975.839	19492.743	0
DPLL	230	4653.228	3460.617	21484.079	0
EP	230	13855.281	9854.502	56547.962	0
IMP	230	7535.673	7265.083	44015.145	0
Output					
DSRM	230	6.774	5.365	24.607	−6.61
DSRW	230	1.454	2.615	11.331	−4.532

#### 4.1.2 Stage 2: environmental variable

In the second phase of analysis, we designate an environmental variable matrix that includes stillbirth rates, alcohol consumption volume, year-on-year changes in the CPI (excluding owner-occupied rent), job availability ratio, unemployment rate, Prefectural Income per Capita^3^ (unit: thousand yen), divorce rate, and marriage rate. These variables are considered factors capable of inducing input slacks, besides random disturbances and managerial inefficiencies. Their descriptive statistics are given in Table 2. Additionally, the data on alcohol consumption is sourced from the National Tax Agency of Japan and the Okinawa Regional Taxation Office, while the remaining data is obtained from publicly available information provided by the Ministry of Health, Labour, and Welfare.

**Table 2:** Descriptive statistics of environmental variables.

	*N*	Mean	SD	Max	Min
Marriage rate	230	4.921	0.551	7.1	3.7
Divorce rate	230	1.795	0.224	2.59	1.33
Unemployment rate	230	3.999	0.888	7.5	2.3
Job availability ratio	230	0.801	0.255	1.57	0.29
Year-on-year changes in the CPI	230	0.512	1.479	4	−1.7
Alcohol consumption volume	230	79.591	10.764	127	60.2
Stillbirth rate	230	23.983	3.102	32.2	17.3
Prefectural Income per Capita	230	2694.174	489.635	5,411	1972

### 4.2 Measurement and correction of efficiency values

#### 4.2.1 Stage 1: initial efficiency scores based on original inputs

This stage employed the SE-MSBM model in the MaxDEA software to evaluate the SPI efficiency across various prefectures in Japan from 2010 to 2014. The efficiency values calculated are presented in Table 3. DMUs with efficiency values less than 1 are considered inefficient in management; those with efficiency values equal to 1 are regarded as efficient and highlighted in yellow in the table; DMUs with efficiency values greater than 1 are considered supper-efficient and marked in red. The last row and column in Table 3 represent the average efficiency values across all DMUs for each year and the average efficiency values for each DMU across all periods, respectively. The evaluation was performed using original inputs and outputs, thus encompassing not only managerial efficiency but also the impact of environmental factors and random noise.

**Table 3:** The initial efficiency score in the Stage 1.

DMUs	2010	2011	2012	2013	2014	Mean
Hokkaido	1.004	0.441	-	1.62	2.366	1.358
Aomori	1.147	1.288	-	1.436	1	1.218
Iwate	0.412	1.225	0.287	0.256	0.145	0.465
Miyagi	1.158	1.913	0.018	0.532	0.406	0.805
Akita	0.223	-	3.012	0.38	0.308	0.981
Yamagata	0.518	0.141	0.044	0.448	0.384	0.307
Fukushima	1.014	1	0.11	0.398	0.381	0.581
Ibaraki	1.008	0.078	0.026	0.592	0.538	0.448
Tochigi	0.678	0.046	0.258	1.171	1.008	0.632
Gunma	1	-	0.022	0.579	0.595	0.549
Saitama	1.018	0.138	0.025	1.018	1.194	0.679
Chiba	1.144	0.069	0.04	1.08	1.091	0.685
Tokyo	1	0.125	0.023	1.163	2.699	1.002
Kanagawa	1.288	0.199	0.069	1.594	1.362	0.903
Niigata	0.38	0.1	0.077	1.081	0.439	0.415
Toyama	1.196	0.098	0.21	0.41	1.077	0.598
Ishikawa	1.206	0.26	0.212	0.458	1.231	0.674
Fukui	0.408	1.206	0.045	0.604	0.599	0.572
Yamanashi	0.495	1	1	1	1.358	0.971
Nagano	0.615	0.172	1	0.552	0.354	0.539
Gifu	1.118	0.052	0.028	1.157	1.292	0.73
Shizuoka	0.603	0.023	1	0.397	1.011	0.607
Aichi	1.081	0.042	0.078	1.682	1.902	0.957
Mie	1.237	0.295	0.017	0.417	0.532	0.5
Shiga	0.463	0.042	1	0.41	0.492	0.481
Kyoto	1.169	0.115	0.037	0.273	0.358	0.391
Osaka	1.105	1.016	0.293	1.007	1	0.884
Hyogo	1.015	0.157	0.019	0.406	0.416	0.403
Nara	0.349	1.013	0.011	0.421	0.328	0.424
Wakayama	1	4.244	1.175	1	1	1.684
Tottori	1.71	1	1.689	4.298	7.458	3.231
Shimane	1.52	1.08	1.165	0.37	0.592	0.946
Okayama	1	0.067	0.044	1.685	0.519	0.663
Hiroshima	1.883	0.234	0.061	1.279	2.414	1.174
Yamaguchi	1.017	0.199	0.215	1.106	1	0.707
Tokushima	0.556	1.683	0.091	0.219	0.146	0.539
Kagawa	0.217	0.018	0.032	0.307	0.415	0.198
Ehime	1.955	0.181	0.085	1.219	1.162	0.92
Kochi	1.051	-	0.284	1	1	0.834
Fukuoka	1.261	0.015	0.038	1.044	0.759	0.623
Saga	0.221	0.063	0.013	0.31	0.33	0.187
Nagasaki	1.265	1.094	1.542	0.625	0.47	0.999
Kumamoto	0.281	0.076	0.058	1.151	1.743	0.662
Oita	0.219	1	0.028	1	1	0.649
Miyazaki	1.407	1.083	0.106	0.53	0.401	0.705
Kagoshima	1.475	0.239	1.043	1.053	1.275	1.017
Okinawa	0.222	0.148	14.067	0.549	0.251	3.047
Mean	0.922	0.561	0.682	0.878	1.017	0.816
S.D.	0.454	0.772	2.129	0.663	1.130	0.574

“-” indicates that the observed values of the DMU for that period are excluded because all of its input indicators are zero, which does not meet the eligibility criteria for the SE-MSBM model.

Overall, the average efficiency across observed DMUs exhibits a U-shaped trend, which starts at 0.922 in 2010, declines rapidly to a minimum of 0.561 by 2011, then steadily rises, reaching a supper-efficient 1.017 by 2014. Moreover, the proportion of DMUs categorized as efficient or supper-efficient compared to all observed DMUs for each period also follows a U-shaped pattern, with percentages of 63.83, 34.09, 24.44, 48.94, and 48.94% for 2010 through 2014, respectively. This indicates an initial decrease followed by an increase in the overall average efficiency of DMUs. On the other hand, the standard deviation of DMU efficiency shows fluctuating trends across periods, reaching a peak of 2.129 in 2012, signifying significant regional disparities in SPI efficiency among prefectures.

A cross-sectional comparison of DMU efficiency over the five-year span reveals that only the average efficiency values of Hokkaido, Aomori, Tokyo, Wakayama, Tottori, Hiroshima, Kagoshima, and Okinawa achieved efficient or supper-efficient levels. Among these, only Wakayama and Tottori consistently achieved efficient or supper-efficient levels throughout the 5 years. This reflects that, even without considering non-managerial factors, hardly any prefecture has an environment or luck factor that readily leads to consistently high-efficiency values.

#### 4.2.2 Stage 2: SFA and OLS

In this stage, the SFA or OLS models are employed to separate the environmental effects and random noise affecting DMU efficiency. Initially, a one-sided error LR test is conducted to test the null hypothesis $H_{0}:\sigma_{u}^{2}=0$. Rejecting this hypothesis indicates that the managerial inefficiency in the input leads to deterministic feasible slack frontiers variations, requiring the use of the SFA model to estimate environmental effects. Failing to reject the null hypothesis suggests that the managerial inefficiency in the input does not affect stochastic frontier variations, indicating that the error term is solely composed of random disturbances, allowing direct estimation using the OLS model. The SFA and OLS models are run using Frontier 2.0 and Stata software, respectively.

According to the results of the LR one-sided error test in Table 4, it’s evident that, at a 95% significance level, we cannot reject the hypothesis of $\sigma_{u}^{2}=0$ for TCSP, EP, and IMP. Therefore, direct estimation using the OLS model is employed. However, for the slack variables of DPLL and PCSP, at a 95% significance level, the hypothesis of $\sigma_{u}^{2}=0$ is rejected, hence employing the SFA model for estimation. Moreover, the *γ* values indicate that managerial inefficiency in these two inputs contributes 43.2 and 56.9% to the error variance, respectively. Additionally, for these two input slacks, we cannot reject the hypothesis of *η* = 0, thus the time invariant SFA model is employed to estimate environmental effects and random influences.

**Table 4:** The results of SFA and OLS in Stage 2.

	SFA		OLS		
	Slack variable of DPLL	Slack variable of PCSP	Slack variable of TCSP	Slack variable of EP	Slack variable of IMP
Marriage rate	566.348 (1.243)	1133.739*** (10.028)	−549.625 (−0.796)	−388.885 (−0.200)	−100.818 (−0.068)
Divorce rate	−188.854 (−0.901)	5501.821*** (84.097)	1,778.137 (1.188)	5,869.685 (1.390)	6,886.430** (2.128)
Unemployment rate	320.415 (0.816)	−255.686 (−0.769)	−726.406* (−1.856)	267.710 (0.242)	299.417 (0.354)
Job availability ratio	−4324.645*** (−21.275)	−3805.223*** (−107.497)	−1,743.026 (−1.340)	−3,725.113 (−1.015)	−440.437 (−0.157)
Year-on-year changes in the CPI	433.096* (2.232)	96.232 (0.478)	−122.107 (−0.754)	938.764** (2.055)	463.961 (1.325)
Alcohol consumption volume	−7.699 (−0.302)	−85.726** (−2.783)	−13.965 (−0.699)	27.424 (0.487)	36.417 (0.843)
Stillbirth rate	−48.600 (−0.628)	−63.606 (−0.668)	18.746 (0.233)	−20.678 (−0.091)	−347.203** (−1.997)
Prefectural Income per Capita	0.789 (1.190)	0.705 (1.371)	0.710 (1.168)	2.286 (1.333)	−0.608 (−0.462)
Constant	−4359.331*** (−43.258)	−8,322.469 (−1.370)	1,318.705 (0.470)	−18,840.891** (−2.378)	−10715.563*** (−3725.296)
$\sigma^{2}$	1.283E+07***	1.971E+07***	8.269E+06***	6.066E+07***	3.752E+07***
LR test of the one-sided error	16.561**	30.082***	6.951	2.631	4.970
γ	0.432***	0.569***	-	-	-
η	−0.091	−0.068	-	-	-

*t*-statistics in parentheses ***p < 0.01, **p < 0.05, *p < 0.1.

As environmental variables affect input slacks inversely to their impact on efficiency values, assessing the influence of environmental variables on efficiency can be done through the sign of regression coefficients. (1) The estimated coefficient for marriage rate is positive, indicating that higher marriage rates tend to lead to slacks in PCSP. Similarly, the positive coefficient for divorce rate suggests that higher divorce rates are associated with slacks in PCSP and IMP. The connection between high divorce rates, high marriage rates, and low efficiency values implies a causal relationship between residents’ marital life entanglements, and the decrease in SPI efficiency. (2) The job availability ratio to some extent reflects the social economic conditions; higher job availability rates correspond to lower slacks in DPLL and PCSP, which favor the improvement of SPI efficiency. (3) CPI positively impacts slacks in EP, suggesting that DMUs with higher CPI exhibit lower SPI efficiency. This reflects a negative correlation between rising living costs and the improvement in SPI efficiency. (4) Alcohol consumption shows a negative correlation with relaxation in PCSP, indicating that DMUs with higher alcohol consumption demonstrate higher SPI efficiency. (5) Stillbirth rates show a negative causal relationship with slacks in IMP, implying that DMUs with higher natural death rates are more likely to enhance SPI efficiency.

#### 4.2.3 Stage 3: final efficiency scores based on adjusted inputs

In the third stage, adjustments were made to the original input indicators based on the regression results from the second stage. Subsequently, the SE-MSBM model was run again using the adjusted input indicators. Table 5 presents the efficiency values of each DMU in the third stage. DMUs with efficiency values greater than 1 are marked in red, while those with efficiency values equal to 1 are marked in yellow.

**Table 5:** The adjusted efficiency score in the Stage 3.

DMUs	2010	2011	2012	2013	2014	Mean
Hokkaido	0.666	1.004	-	1.299	1.233	1.05
Aomori	1.106	1.174	-	2.064	1	1.336
Iwate	0.271	1.114	0.285	0.208	0.11	0.398
Miyagi	0.414	1.087	0.445	0.439	0.281	0.533
Akita	0.181	-	1.156	0.253	0.221	0.453
Yamagata	0.359	0.405	0.35	0.324	0.271	0.342
Fukushima	0.588	1.156	0.425	0.291	0.258	0.544
Ibaraki	0.658	0.392	0.461	0.507	0.431	0.49
Tochigi	0.499	0.436	0.576	0.673	0.461	0.529
Gunma	0.385	-	0.38	0.467	0.388	0.405
Saitama	0.537	0.461	0.521	1	1.061	0.716
Chiba	1.063	0.436	0.5	0.797	0.686	0.697
Tokyo	1	0.464	0.521	1.047	1.251	0.857
Kanagawa	1.488	0.504	0.617	1.005	1.342	0.991
Niigata	0.239	0.492	0.449	0.522	0.228	0.386
Toyama	1.139	0.381	0.486	0.367	0.417	0.558
Ishikawa	0.321	0.503	0.541	0.436	0.453	0.451
Fukui	0.344	1.042	0.364	0.439	0.364	0.511
Yamanashi	0.347	1	1	1	1.582	0.986
Nagano	0.426	0.453	1.075	0.415	0.241	0.522
Gifu	0.623	0.401	0.374	0.514	0.734	0.529
Shizuoka	0.376	0.376	1.025	0.367	0.671	0.563
Aichi	0.411	0.346	0.488	1.214	1.24	0.74
Mie	1.571	0.533	0.416	0.417	0.362	0.66
Shiga	0.207	0.345	1.022	0.4	0.334	0.461
Kyoto	0.497	0.446	0.422	0.311	0.342	0.404
Osaka	1.01	1.005	0.719	1	1	0.947
Hyogo	0.496	0.58	0.512	0.409	0.271	0.454
Nara	0.25	0.645	0.342	0.345	0.243	0.365
Wakayama	1	4.388	1.194	1	1	1.716
Tottori	1	1	1.231	4.434	2.097	1.952
Shimane	1.346	1.025	1.004	0.244	0.287	0.781
Okayama	1.386	0.496	0.534	0.593	0.383	0.678
Hiroshima	1.684	0.468	0.538	1.051	0.62	0.872
Yamaguchi	1	0.399	0.485	1	1	0.777
Tokushima	0.493	1.494	0.495	0.17	0.114	0.553
Kagawa	0.239	0.308	0.408	0.283	0.236	0.295
Ehime	2.05	0.469	0.478	1.038	0.489	0.905
Kochi	0.353	-	0.558	0.384	0.286	0.396
Fukuoka	1.533	0.446	0.575	0.655	0.51	0.744
Saga	0.181	0.32	0.322	0.295	0.248	0.273
Nagasaki	0.626	1.016	1.131	0.504	0.312	0.718
Kumamoto	0.224	0.405	0.463	0.575	1.036	0.54
Oita	0.22	0.56	0.487	0.529	0.3	0.419
Miyazaki	1.465	0.506	0.482	0.421	0.271	0.629
Kagoshima	1.165	0.545	0.658	0.65	1.027	0.809
Okinawa	0.182	0.34	1.183	0.336	0.179	0.444
Mean	0.715	0.713	0.616	0.696	0.593	0.666
S.D.	0.491	0.644	0.278	0.668	0.444	0.335

“-” indicates that the observed values of the DMU for that period are excluded because all of its input indicators are zero, which does not meet the eligibility criteria for the SE-MSBM model.

The adjustments to the original input indicators in the second stage subjected all DMUs to the worst external environmental and luck-related factors, consequently leading to a decrease in efficiency values for most DMUs. Specifically, after removing the influences of external environment and luck-related factors, the number of DMUs with 5-year average efficiency values greater than or equal to 1 decreased from 8 in the first stage to 4, including Hokkaido, Aomori, Wakayama, and Tottori. Furthermore, compared to the first stage, 61.7% of DMUs experienced a decline in their average efficiency values over the 5-year period. However, despite the removal of external environmental and luck-related factors, Wakayama and Tottori continued to maintain efficient or super-efficient level over the 5-year period.

In contrast to the U-shaped average efficiency trend across all observed DMUs in the first stage, the average efficiency of DMUs displayed a fluctuating downward trend after the removal of external environmental and luck-related factors. Starting from its peak of 0.715 in 2010, the average efficiency demonstrated a steady decline, reaching 0.593 by 2014, with a minor uptick in 2013. This indicates that, at least during the ascending phase (2011 to 2014) of the U-shaped average efficiency trend observed in the first stage, favorable external environmental and luck-related factors masked the fact of low managerial efficiency among DMUs, resulting in a rebound trend in average efficiency. Additionally, compared to the first stage, the standard deviation of efficiency values for DMUs across periods tended to stabilize, indicating that external environmental and luck-related factors increased regional disparities in the efficiency of SPI among DMUs.

### 4.3 Dynamic evolution of suicide prevention efficiency

For the specific causes of SPI efficiency’s variation, a further dynamic decomposition is necessary. Utilizing the MaxDEA software, this study employs the adjacent LTFP analysis to examine the relative shifts between prefectures and the production frontier, wherein LTFP, LPEC, LSEC, LPTP, and LTPSC, respectively, denote total factor productivity, pure efficiency changes, scale efficiency changes, pure productivity technology changes, and technology scale changes. It’s important to note that in contrast to interpreting results from SE-MSBM, if LTFP and its decomposition are greater than 0, it signifies an improvement in productivity, efficiency, or technology compared to the base period; conversely, if it’s less than 0, it indicates a decline.

Table 6 displays the average LTFP and its decomposition for all observed DMUs from 2010 to 2014. From Table 6, we observe that the national LTFP is positive from 2010 to 2013, indicating an overall increase in SPI productivity. However, the reasons for this productivity increase differ. The rise in LTFP from 2010 to 2011 is attributed to the enhancement of LSEC, leading to an improvement in LEC. Conversely, the regression in LTPSC diminished the magnitude of the productivity increase during that period. Improvements in productivity from 2011 to 2012 and 2012 to 2013 are credited to LTC. The former is due to improvements in LPTP, while the latter stems from the enhancement of LTPSC. Lastly, from 2013 to 2014, both LPTP and LTPSC exhibit notable improvements. However, the decline in LPEC and LSEC is greater, resulting in a slight regression in productivity.

**Table 6:** Luenberger total factor productivity and its composition.

Period	LTFP						
		LEC			LTC		
			LPEC	LSEC		LPTP	LTPSC
2010–2011	0.903	2.084	−0.03	2.114	−1.18	0.204	−1.384
2011–2012	0.131	−0.042	−0.085	0.043	0.146	0.233	−0.087
2012–2013	0.170	−0.534	0.036	−0.570	0.704	−0.21	0.914
2013–2014	−0.052	−0.874	−0.103	−0.771	0.798	0.383	0.415
Mean	0.283	0.139	−0.046	0.185	0.123	0.149	−0.026

Overall, over the five-year period from 2010 to 2014, the average SPI productivity across Japan increased by 28.3%. Among this, 13.9% is attributed to the progress of LEC, while 12.3% is credited to LTC’s advancements. Notably, the majority of LEC’s progress comes from the enhancement of LSEC, while the advancements of LTC overall benefits from the progress of LPTP. Furthermore, in terms of developmental trends, LEC has exhibited a gradual decline since 2011, with the rate of decrease intensifying each year. This decline primarily stems from the overall regression of LSEC, which has shown a progressively increasing downward trend, while the contribution from changes in LPEC remains relatively limited. Conversely, although LTC displayed a significant decline from 2010 to 2011, it has since stabilized and shown a consistent upward trend, with the rate of increase intensifying each year. Both LPTP and LTPSC have made significant contributions to the changes observed in LTC from 2011 onward.

### 4.4 Robustness test

The concern regarding the robustness of this study originates from the insignificance of the OLS regression in the second stage SE-MSBM. Despite Fried, Lovell (32)‘s indication that under the circumstances where the LR one-sided error test fails to reject the null hypothesis $H_{0}:\sigma_{u}^{2}=0$, the stochastic frontier specification is rejected, hence obviating the need for the SFA model. However, the OLS regression in Table 4 demonstrates less significant estimation effects for environmental factor coefficients. As a robustness check, this study revisits the estimation of environmental factors’ contribution on the slacks in TCSP, EP, and IMP in the second stage, using the SFA model to estimate the coefficients instead of the OLS, and subsequently adjusts the input indicators with the estimated coefficients from SFA. As shown in Table 7, compared to OLS, the results from SFA exhibit greater significance, indicating an enhanced explanatory power of environmental variables on the slacks of TCSP, EP, and IMP after accounting for managerial inefficiency’s impact.

**Table 7:** The results of SFA for robustness test.

	Slack variable of TCSP	Slack variable of EP	Slack variable of IMP
Marriage rate	−540.976*** (−43.829)	−382.821*** (−25.485)	−60.352 (−0.620)
Divorce rate	1777.163*** (166.431)	5872.930*** (722.722)	6889.457*** (1003.856)
Unemployment rate	−683.387*** (−9.422)	284.469*** (7.021)	407.418 (1.529)
Job availability ratio	−1756.093*** (−76.788)	−3728.465*** (−476.787)	−456.843*** (−11.125)
Year-on-year changes in the CPI	−207.760 (−1.555)	921.354*** (22.935)	408.200** (2.930)
Alcohol consumption volume	−17.924 (−0.919)	−1.732 (−0.031)	11.519 (0.266)
Stillbirth rate	29.615 (0.565)	37.096 (0.258)	−324.267** (−2.708)
Prefectural Income per Capita	0.734* (2.164)	2.629** (2.767)	−0.255 (−0.383)
Constant	1,318.705 (0.470)	−21401.608*** (−5296.289)	−10715.563*** (−3725.296)
$\sigma^{2}$	8.269E+06***	6.066E+07***	3.752E+07***
LR test of the one-sided error	6.951	2.631	4.970
γ	-	-	-
η	−0.114	−0.048	−0.028

*t*-statistics in parentheses ***p < 0.01, **p < 0.05, *p < 0.1.

Following adjustments to the related input variables based on estimated coefficients, the third stage of the SE-MSBM model is rerun. The efficiency values estimated are then utilized for a subsequent adjacent LTFP analysis, as presented in Table 8. Contrasting the results obtained from Table 6, the most notable change observed after considering managerial inefficiency in certain inputs is the shift of the 2013–2014 LTFP from negative to positive. This reversal is attributed to the absolute difference between positive LTC and negative LEC. Furthermore, for other periods and the overall productivity coefficients, the results of the robustness test exhibit minimal discrepancies from the primary analysis, signifying the robustness of the original analytical outcomes.

**Table 8:** Luenberger total factor productivity and its composition for robustness test.

Period	LTFP						
		LEC			LTC		
			LPEC	LSEC		LPTP	LTPSC
2010–2011	0.933	2.111	−0.034	2.145	−1.178	0.219	−1.397
2011–2012	0.132	−0.043	−0.085	0.042	0.148	0.245	−0.097
2012–2013	0.153	−0.565	0.054	−0.619	0.719	−0.224	0.943
2013–2014	0.157	−0.248	−0.112	−0.136	0.363	0.381	−0.018
Mean	0.342	0.303	−0.045	0.348	0.015	0.152	−0.137

## 5 Discussion

In the three-stage SE-MSBM model, the results from the first stage reflect the SPI efficiency of each prefecture under their respective environmental and stochastic influences. The outcomes from the third stage represent the relative actual managerial efficiency of each prefecture after removing the effects of environmental and stochastic factors. Comparing the results from the third stage with those from the first stage, it’s observed that the differences in suicide prevention efficiency among prefectures are more pronounced in the first stage. This suggests that the uneven distribution of external environmental and stochastic factors amplifies regional disparities in SPI efficiency among prefectures. From another perspective, the differences in suicide prevention efficiency observed in reality, attributable solely to managerial efficiency across prefectures, only constitute a portion of the overall observed differences. Neglecting the influence of exogenous factors might potentially exaggerate spatial discrepancies in SPI efficiency.

The analysis concerning the impact of environmental factors on input slack in the second stage has revealed some intriguing findings. (1) Both marriage rates and divorce rates are positively correlated with input slack (negatively associated with efficiency values). Assuming that the societal institution of marriage has a protective effect on suicide prevention (17, 18), higher marriage rates and lower divorce rates should result in decreased suicide rates, thereby enhancing SPI efficiency. However, the negative correlation between marriage rates and efficiency values suggests that married life could potentially serve as a trigger for higher suicide rates. According to publicly available data from the Japan National Police Agency (41), family issues remained the second leading cause of female suicide after health issues during this period, and approximately 10% of male suicides were attributable to family issues. Thus, entering into marriage may offer some protection against suicide on one hand, while on the other hand, it may introduce additional stressors, thereby increasing suicide risk. Moreover, the impact of marriage rates on input slack via the PCSP underscores the significant role of “seeking help” in reducing the suicide rate among individuals facing marital and family problems. Inefficient management and utilization of funds in this sector might lead to an increase in suicides related to marital issues, consequently affecting the overall SPI efficiency of the DMUs. (2) The negative correlation between the job availability ratio and input slack implies that a robust economic environment is more conducive to improving SPI efficiency. Economic and livelihood issues were the second leading cause of male suicides in Japan between 2009 and 2014 (41). Additionally, the surge in Japan’s suicide rate in 1998 is believed to be causally linked to economic downturns (20, 42). Therefore, in terms of enhancing SPI efficiency, prefectures facing poorer economic conditions encounter stronger resistance. (3) The negative correlation between CPI and efficiency suggests that increased pressure from higher prices may elevate the risk of suicide, consequently impeding improvements in the effectiveness of SPI. (4) Elevated alcohol consumption tends to decrease the slacks of PCSP, subsequently enhancing efficiency. Despite the common belief that high alcohol consumption increases the risk of suicide (15, 21, 43), considering the impact of alcohol consumption on PCSP slacks and its potential for alleviating tension and anxiety (44), it is speculated that individuals may be more inclined to engage in conversations (‘talk it out’) post-alcohol consumption, thus paradoxically reducing the risk of suicide to some extent. However, conflicting evidence in prior studies necessitates further examination of the mediating effects between alcohol consumption and the effectiveness of SPI. (5) There exists a positive correlation between the stillbirth rate and efficiency. Previous studies commonly assert that the stillbirth rate increases the risk of female suicide, which contradicts the findings here. One plausible explanation for this outcome could be, according to the Guidelines for the Management and Operation of the Regional Suicide Prevention Emergency Strengthening Fund (8), the IMP project serves as a safety net, wherein funds that cannot be allocated to other projects are designated to IMP. Therefore, the causal relationship between the stillbirth rate and the IMP slacks likely attributes to the funding division method of RF.

The analysis of the LTFP index indicates an overall upward trend in SPI productivity across Japan, despite a slight setback observed in 2013–2014. Additionally, there is a declining trend in the marginal improvement of productivity. Although the contributions of LEC and LTC to productivity improvement seem similar based on the outcomes, the developmental trends since 2011 reveal a gradual deterioration in LEC, contrasted with a gradual improvement in LTC. Moreover, the absolute values of the marginal change rates for both indicators have been steadily increasing. This indicates that post-2011, the overall improvement in SPI productivity of DMUs relied predominantly on technological enhancements, and technological advancements masked the deterioration in efficiency. Focusing on the causes of efficiency decline, it becomes evident that due to limited fluctuations in LPEC, the deteriorating trend in LSEC should primarily shoulder the responsibility.

We consider that the 2011 Great East Japan Earthquake has certain connections with the SPI efficiency across Japan. Occurring on March 11, 2011, this natural disaster is considered to have affected residents’ psychological and mental health, increasing the risk of suicide (11, 45, 46). According to nationwide studies, female suicide rates surged rapidly in the months following the earthquake, whereas male suicide rates, especially among middle-aged and older men, notably decreased in the months following the earthquake, although the declining trend weakened over time (45, 47). The contrasting trends in male and female suicide rates posed challenges for prefectural SPI efforts. Furthermore, in the 2 years following the earthquake, the proportion of female suicides attributed to “health issues” gradually increased (48). According to the findings of Kato and Okada (5), female suicide rates were inversely proportional to the amount of DPLL at the municipal level in the RF. Therefore, the post-2011 improvement in the management and utilization efficiency of DPLL might have contributed more to the efficiency enhancement. Lastly, the Great East Japan Earthquake might be an important exogenous environmental factor influencing SPI efficiency across prefectures. However, due to the inability to accurately delineate the effects of the natural disaster and its spillover effects on residents’ suicide risk, this study could not precisely control for its impact on DMU efficiency, suggesting room for improvement in future research.

## 6 Conclusion

Existing studies on the decline in suicide rates in Japan from 2009 to 2014 have predominantly focused solely on the changes in suicide rates, overlooking the fiscal costs incurred by the government in controlling these rates. In the context of severe financial constraints, this outcome-oriented research approach has led to a lack of understanding regarding the cost-effectiveness of policies, consequently detaching the assessment of policy effectiveness from practical implications. Thus, this study concentrates on the cost-effectiveness of SPI by prefectural governments in Japan from 2009 to 2014, marking the first application of the DEA analysis framework to evaluate the government’s efficiency in SPI.

Specifically, we employed the SE-MSBM model, using the declared amounts of RF as inputs and the reduction in male and female suicide rates as outputs to assess the efficiency of SPI across Japanese prefectures. Furthermore, to eliminate the influence of exogenous environmental factors and luck on efficiency, we utilized a three-stage analysis framework to derive a relatively objective managerial efficiency of SPI in these prefectures. Subsequently, to elucidate the dynamic changes in efficiency and their causes, we employed the LTFP index and its decomposition to analyze the productivity changes in SPI efficiency.

The analysis results indicate the following: Firstly, in the three-stage SE-MSBM analysis, the spatial differences in SPI efficiency reflected in the results of the third stage are relatively minor compared to those in the first stage. This suggests that efficiency values directly based on inputs and outputs are easily influenced by exogenous environmental and luck factors. However, after filtering out these exogenous factors, the differences in efficiency values resulting from the managerial efficiency disparities among prefectures are relatively limited. Secondly, the analysis in the second stage of the impact of environmental and luck factors on efficiency values reveals that high divorce rates, high marriage rates, high year-on-year changes in the CPI, low job availability ratio, and low alcohol consumption are likely to hinder the improvement of SPI efficiency. Finally, in the analysis of the LTFP index, we found that although the overall average productivity of SPI in prefectures maintains an upward trend, the marginal improvement demonstrates a diminishing trend. Additionally, productivity improvement comprises positive technical changes and negative efficiency changes, with the negative efficiency changes primarily attributable to the continuous deterioration of scale efficiency.

The results of this study provide some insights for policymakers: Firstly, in the process of enhancing the efficiency of SPI, efforts should not only focus on improving management efficiency but also consider improving the external environment. Secondly, an excessive reliance on technical advancements is observed in the improvement of SPI productivity, insufficiently supported by productivity enhancements arising from efficiency improvements. This highlights the need for further optimization of policy execution efficiency in each prefecture, ensuring that resources are used efficiently. Lastly, optimizing scale efficiency to promote positive efficiency changes, thereby enhancing SPI productivity, appears to be the most evident approach.

We must acknowledge several limitations in this study. Firstly, in the selection of environmental variables, our approach was to include external factors influencing input–output and factors affecting input–output conversion rates in the environmental variable matrix. However, the declaration rules of RF generally did not have specific limitations based on the external environment of each prefecture. Moreover, the input–output conversion rates mainly reflect the fund management and utilization efficiency of DMUs, which is precisely the efficiency that the three-stage SE-MSBM model aims to measure. Therefore, most variables eventually considered as environmental factors tend to exert exogenous influences on output variables, namely male and female suicide rates. The limitation of this variable selection approach lies in the possibility that if other exogenous environmental variables exist, the second stage’s filtering effect on environmental and stochastic factors might be inadequate. Secondly, considering that RF does not subsidize SPI policies that had received other subsidies before 2009, we assumed that relevant policies implemented before 2009 continued unchanged during the study period. If this assumption does not hold, the reduction in suicide rates following RF implementation might contain confounding factors. Further examinations and analyses would rely on more precise data support.

## Data Availability

The original contributions presented in the study are included in the article/Supplementary material, further inquiries can be directed to the corresponding author.

## Footnotes

^1^The RF was allocated 10 billion yen in the supplementary budget for the 2009 fiscal year, 3.7 billion yen in the third supplementary budget for the 2021 fiscal year, 30.2 billion yen in the first supplementary budget for the 2022 fiscal year, and 16.3 billion yen in the first supplementary budget for the 2023 fiscal year.

^2^The RF data utilized in this study is the sum of the portion directly requested by prefectural governments from the central government and the portion representing funding usage plans submitted by municipalities within the jurisdiction of the prefectures.

^3^Based on 2011 standard.

## References

[1] World Health Organization (2014). Preventing suicide: a global imperative.

[2] World Health Organization (2021). Comprehensive mental health action plan 2013–2030.

[3] Cabinet Office, Government of Japan (2014). White paper on suicide prevention in Japan: 2014 edition (Heisei 26). Tokyo, Japan.

[4] Takeshima T Yamauchi T Inagaki M Kodaka M Matsumoto T Kawano K. Suicide prevention strategies in Japan: a 15-year review (1998-2013). J Public Health Policy. (2015) 36:52–66. doi: 10.1057/jphp.2014.42, PMID: 25355234

[5] Kato R Okada M. Can financial support reduce suicide mortality rates? Int J Environ Res Public Health. (2019) 16:4797. doi: 10.3390/ijerph16234797, PMID: 31795379 PMC6926693

[6] Okada M Hasegawa T Kato R Shiroyama T. Analysing regional unemployment rates, GDP per capita and financial support for regional suicide prevention programme on suicide mortality in Japan using governmental statistical data. BMJ Open. (2020) 10:e037537. doi: 10.1136/bmjopen-2020-037537, PMID: 32859665 PMC7454243

[7] Ministry of Health, Labour and Welfare (2012). Evaluation report on the regional comprehensive suicide prevention emergency strengthening fund.

[8] Ministry of Health, Labour and Welfare (2009). Guidelines for the management and operation of the regional suicide prevention emergency strengthening fund.

[9] Nakano T Hasegawa T Okada M. Analysing the impacts of financial support for regional suicide prevention Programmes on suicide mortality caused by major suicide motives in Japan using statistical government data. Int J Environ Res Public Health. (2021) 18:3414. doi: 10.3390/ijerph18073414, PMID: 33806105 PMC8036759

[10] Nakanishi M Yamauchi T Takeshima T. National strategy for suicide prevention in Japan: impact of a national fund on progress of developing systems for suicide prevention and implementing initiatives among local authorities. Psychiatry Clin Neurosci. (2015) 69:55–64. doi: 10.1111/pcn.12222, PMID: 25041482

[11] Nakanishi M Endo K Ando S Nishida A. The impact of suicide prevention act (2006) on suicides in Japan. Crisis. (2020) 41:24–31. doi: 10.1027/0227-5910/a000599, PMID: 31066309

[12] Inoue K Nishimura Y Okazazi Y Fukunaga T. Discussion based on analysis of the suicide rate and the average disposable income per household in Japan. West Indian Med J. (2014) 63:340–3. doi: 10.7727/wimj.2012.298, PMID: 25429478 PMC4663925

[13] Shiroyama T Fukuyama K Okada M. Effects of financial expenditure of prefectures/municipalities on regional suicide mortality in Japan. Int J Environ Res Public Health. (2021) 18:8639. doi: 10.3390/ijerph18168639, PMID: 34444387 PMC8394344

[14] Yonemoto N Kawashima Y Endo K Yamada M. Implementation of gatekeeper training programs for suicide prevention in Japan: a systematic review. Int J Ment Heal Syst. (2019) 13:2. doi: 10.1186/s13033-018-0258-3, PMID: 30622628 PMC6317190

[15] Stack S. Contributing factors to suicide: political, social, cultural and economic. Prev Med. (2021) 152:106498. doi: 10.1016/j.ypmed.2021.106498, PMID: 34538366

[16] Flavin P Radcliff B. Public policies and suicide rates in the American states. Soc Indic Res. (2008) 90:195–209. doi: 10.1007/s11205-008-9252-5, PMID: 39692860

[17] Fujimoto M Yokoyama K. Family ties: exploring suicide using prefecture-level panel data. Oikonomika. (2016) 52:1–15.

[18] Fukuchi N Kakizaki M Sugawara Y Tanji F Watanabe I Fukao A. Association of marital status with the incidence of suicide: a population-based cohort study in Japan (Miyagi cohort study). J Affect Disord. (2013) 150:879–85. doi: 10.1016/j.jad.2013.05.006, PMID: 23830860

[19] Motillon-Toudic C Walter M Séguin M Carrier JD Berrouiguet S Lemey C. Social isolation and suicide risk: literature review and perspectives. Eur Psychiatry. (2022) 65:e65. doi: 10.1192/j.eurpsy.2022.2320, PMID: 36216777 PMC9641655

[20] Nakanishi M Endo K. National Suicide Prevention, local mental health resources, and suicide rates in Japan. Crisis. (2017) 38:384–92. doi: 10.1027/0227-5910/a000469, PMID: 28748710

[21] Brady J. The association between alcohol misuse and suicidal behaviour. Alcohol Alcohol. (2006) 41:473–8. doi: 10.1093/alcalc/agl060, PMID: 16891335

[22] Russell R Metraux D Tohen M. Cultural influences on suicide in Japan. Psychiatry Clin Neurosci. (2017) 71:2–5. doi: 10.1111/pcn.12428, PMID: 27487762

[23] Kuroki M. Suicide and unemployment in Japan: evidence from municipal level suicide rates and age-specific suicide rates. J Socio-Econ. (2010) 39:683–91. doi: 10.1016/j.socec.2010.06.009

[24] Huang C-J Ho Y-H. Does high unemployment rate cause high suicide rate? Evidence from Japan and South Korea. J Rev Glob Econ. (2016) 5:165–70. doi: 10.6000/1929-7092.2016.05.14

[25] Erdem C Dinç M. The socioeconomic determinants of suicide: a panel data analysis. Yildiz Soc Sci Rev. (2022) 8:1–12. doi: 10.51803/yssr.1146860

[26] Yosano A. Proposal of a novel strategy for identifying independent variables for causal analysis of suicide rates: pilot study applying self-organizing maps. Trust Soc. (2023) 3:1–28.

[27] Mbau R Musiega A Nyawira L Tsofa B Mulwa A Molyneux S. Analysing the efficiency of health systems: a systematic review of the literature. Appl Health Econ Health Policy. (2023) 21:205–24. doi: 10.1007/s40258-022-00785-2, PMID: 36575334 PMC9931792

[28] Dunlap LJ Orme S Zarkin GA Arias SA Miller IW Camargo CA Jr. Screening and intervention for suicide prevention: a cost-effectiveness analysis of the ED-SAFE interventions. Psychiatr Serv. (2019) 70:1082–7. doi: 10.1176/appi.ps.201800445, PMID: 31451063 PMC12051401

[29] Linskens EJ Venables NC Gustavson AM Sayer NA Murdoch M MacDonald R. Population- and community-based interventions to prevent suicide. Crisis. (2023) 44:330–40. doi: 10.1027/0227-5910/a000873, PMID: 36052582

[30] Tone K. A slacks-based measure of super-efficiency in data envelopment analysis. Eur J Oper Res. (2002) 143:32–41. doi: 10.1016/S0377-2217(01)00324-1

[31] Sharp JA Meng W Liu W. A modified slacks-based measure model for data envelopment analysis with ‘natural’ negative outputs and inputs. J Oper Res Soc. (2017) 58:1672–7. doi: 10.1057/palgrave.jors.2602318

[32] Fried HO Lovell CAK Schmidt SS Yaisawarng S. Accounting for environmental effects and statistical noise in data envelopment analysis. J Prod Anal. (2002) 17:157–74. doi: 10.1023/A:1013548723393

[33] Guan HJ Wang Y Dong LY Zhao AW. Efficiency decomposition analysis of the marine ship industry chain based on three-stage super-efficiency SBM model-evidence from Chinese A-share-listed companies. Sustain For. (2022) 14:2155. doi: 10.3390/su141912155

[34] Chen YD Li CH Li XX Zhang XL Tan Q. Efficiency of water pollution control based on a three-stage SBM-DEA model. Water. (2022) 14:1453. doi: 10.3390/w14091453

[35] Chen Y Ma L Zhu Z. The environmental-adjusted energy efficiency of China's construction industry: a three-stage undesirable SBM-DEA model. Environ Sci Pollut Res Int. (2021) 28:58442–55. doi: 10.1007/s11356-021-14728-2, PMID: 34114142

[36] Jondrow J Lovell CAK Materov IS Schmidt P. On the estimation of technical inefficiency in the stochastic frontier production function model. J Econ. (1982) 19:233–8. doi: 10.1016/0304-4076(82)90004-5, PMID: 39185140

[37] Fang Z Jiang L Fang Z. Does economic policy intervention inhibit the efficiency of China’s green energy economy? Sustain For. (2021) 13:3412. doi: 10.3390/su132313412

[38] Chambers RG Fāure R Grosskopf S. Productivity growth in Apec countries. Pac Econ Rev. (2007) 1:181–90. doi: 10.1111/j.1468-0106.1996.tb00184.x, PMID: 39675774

[39] Grosskopf S. Some remarks on productivity and its decompositions. J Prod Anal. (2003) 20:459–74. doi: 10.1023/A:1027364119672

[40] Cantor VJM Poh KL. Integrated analysis of healthcare efficiency: a systematic review. J Med Syst. (2017) 42:8. doi: 10.1007/s10916-017-0848-7, PMID: 29167999

[41] Suicide Statistics. Fundamental data on suicide in the region [internet]. Japan National Police Agency. (2014). Available at: https://www.mhlw.go.jp/stf/seisakunitsuite/bunya/0000140901.html (Accessed January 12, 2023).

[42] Okamura K Ikeshita K Kimoto S Makinodan M Kishimoto T. Suicide prevention in Japan: government and community measures, and high-risk interventions. Asia Pac Psychiatry. (2021) 13:e12471. doi: 10.1111/appy.12471, PMID: 33787084

[43] Sher L. Alcohol consumption and suicide. QJM. (2006) 99:57–61. doi: 10.1093/qjmed/hci146, PMID: 16287907

[44] Abrams KB Mei L Chen CS Koele E Kwan J Montes Z. The paradoxical association between tension-reduction alcohol outcome expectancies and tension following alcohol consumption. Am J Drug Alcohol Abuse. (2022) 48:206–16. doi: 10.1080/00952990.2021.1992772, PMID: 34781788

[45] Orui M. Suicide and suicide prevention activities following the great East Japan earthquake 2011: a literature review. Int J Environ Res Public Health. (2022) 19:906. doi: 10.3390/ijerph191710906, PMID: 36078620 PMC9518051

[46] Suzuki Y Kim Y. The great East Japan earthquake in 2011; toward sustainable mental health care system. Epidemiol Psychiatr Sci. (2012) 21:7–11. doi: 10.1017/S2045796011000795, PMID: 22670406

[47] Osaki Y Otsuki H Imamoto A Kinjo A Fujii M Kuwabara Y. Suicide rates during social crises: changes in the suicide rate in Japan after the great East Japan earthquake and during the COVID-19 pandemic. J Psychiatr Res. (2021) 140:39–44. doi: 10.1016/j.jpsychires.2021.05.035, PMID: 34090102 PMC8674964

[48] Inoue K Fujita Y Miyaoka T Ezoe S Horiguchi J. Importance of measures to prevent suicides related to the great East Japan earthquake among women. Psychiatry Clin Neurosci. (2015) 69:596. doi: 10.1111/pcn.12288, PMID: 25756605

